# On Having a Goal: Goals as Representations or Behavior

**DOI:** 10.1007/s40732-014-0093-0

**Published:** 2014-08-28

**Authors:** Jonas Ramnerö, Niklas Törneke

**Affiliations:** 1 Department of Psychology, Stockholm University, S106 91 Stockholm, Sweden; 2 Kalmar, Sweden

**Keywords:** Goals, Goal-directed behavior, Rule-governed behavior, Relational frame theory

## Abstract

The present article discusses the concepts of *having a goal* and of *goal-directed behavior* from a behavior-analytic perspective. In clinical psychology as well as in the study of human behavior at large, goals delineate an important area of investigation when it comes to health, well-being, and behavioral change. While concepts like goals and goal-directed behavior may be more frequently used outside the theoretical boundaries of behavior analysis, we argue that by incorporating recent behavior analytic research on verbal behavior, new and fruitful ways open up for approaching the phenomenon of *having a goal*. A behavior-analytic approach thereby may increase both precision in understanding and the potential for influencing essential aspects of human behavior. This analysis starts with the concept of rule-governed behavior and develops that analysis by using the concept of derived relational responding.

To “have a goal” is probably a universal experience in language-able humans, and the custom of talking about one’s goals is a commonplace behavior in everyday conversation. Future goals tend to be ascribed significant value, on a personal basis, and goal-oriented behavior almost seems to delineate the complexity of human responding in comparison with that of other species. Clinicians who reside within a behavioral tradition are trained to emphasize clear and tangible goals in treatment. This approach seems to make a lot of sense to patients, students, and clinicians. Individuals are in a sense impregnated with the idea that their personal goals are pivotal in terms of forming a valuable life. From a theoretical point of view, the concept of goals has also acquired a central role in psychology as a means of explaining behavior. This role dates back to the 1960s, when the dramatic shift toward cognitive theories occurred and the concept of needs was replaced by the concept of goals as a dominant motivational concept (Deci and Ryan 2000).

At the same time, there is a relative paucity of behavior-analytic accounts of goal-directed behavior. This situation may not be very surprising in that goals (as humans tend to perceive them) are temporarily located in a future yet to exist. The commonsense term *goal-directed behavior* thereby seems to defy experimental logic, where causes must precede the behavior to be explained, and the term thereby runs into problems within a psychological framework that stresses experimentally verified principles. Behavior analysis emphasizes clear variables that are amenable to deliberate influence in order to pave the way for scientific progress in areas where the language of folk psychology is dominant. Outside the behavior-analytic tradition, the concept of goals is used extensively, and, as it seems, with good reason. The empirical research that has emanated from the concept of goals does seem to delineate something of vital importance to human beings. Before we attempt to articulate a behavioral conceptualization of goal-directed behavior, we will go through some of the research on goals of particular relevance for clinical practice. In order to do that, we will momentarily set aside the definitional issues and instead rely on the reader’s commonsense understanding of the term *goal*, and then return to the problems inherent in the commonsense notion of *having a goal*.

## An Overview of Empirical Findings on Goals

### Goals as Related to Personal Functioning

A number of basic conceptual dimensions regarding goals have been proposed and have generated interesting empirical findings. A distinction often made is that between intrinsic and extrinsic goals (Kasser and Ryan 1993, 1996). Intrinsic goals are those pertaining to affiliation, personal growth, and community contribution, which are assumed to be closely related to the satisfaction of what Kasser and Ryan referred to as basic human needs, whereas extrinsic goals are those concerning the attainment of wealth, fame, and factors more related to external signs of worth. Extrinsic goals are assumed to be less related to basic-need satisfaction and may even distract from this process. The literature is replete with studies stating that having clear and valued goals is related to positive psychological functioning (e.g., Brunstein 1993; Emmons 1992; Sheldon and Elliot 1999). What would be labeled as an orientation toward extrinsic rather than intrinsic goals has been shown to be associated with increased risk on variables such as the use of tobacco and drugs (Williams et al. 2000). Furthermore, endorsement of extrinsic goals, in comparison with intrinsic goals, tends to be correlated with depression, anxiety, and negative affect among other variables (Kasser and Ryan 1993, 1996). People who report an extrinsic goal orientation also tend to report having been raised with less nurturing child-raising styles—that is, styles that are more controlling than supportive (Kasser et al. 1995).

Another health-relevant goal dimension is whether goals are approach or avoidance oriented. Candidates for psychotherapy have been found to be more oriented toward avoidance goals than are normal controls, and intensity of orientation toward avoidance goals correlates with severity of psychopathology, poor well-being, and other psychological problems in psychotherapy patients as well as in normal controls (Grosse Holtforth and Grawe 2002). Cox and Klinger (2002) approached what was seen as superordinate aspects of different goal-related constructs in terms of structure, where an adaptive goal structure is characterized by the person’s ability to formulate approach goals that he or she is committed to, where the fulfillment of these goals is emotionally rewarding, and where he or she is optimistic regarding the possibility of goal achievement. What was labeled a maladaptive goal structure lacks one or several of these aspects. Maladaptive goal structure in combination with high perceived task difficulty and stress has been found to be associated with depression among students (Lecci et al. 1994).

In a clinical setting, Pöhlmann (as cited in Grosse Holtforth and Grawe 2002) found that patients with psychosomatic disorders tended to endorse a higher number of goals than healthy controls did. However, the patients’ goals were dominated by avoidance orientation and often formulated within a narrow area of concern. Also, within a sample of patients treated for anxiety disorders, stating conflicting personal goals showed negative correlation with therapeutic behaviors such as cooperation, openness, and readiness to try new behaviors but were positively correlated to resistance and a tendency to drop out (Michalak et al. 2004).

### Goals as Related to Therapy and Behavior Change

Several important functions have been attributed to the concept of goals within the area of psychological treatments. For example, goals provide attentional focus for both client and therapist and provide criteria for evaluation of progress. There is also the possibility that the process of formulating goals exerts a therapeutic effect in itself (Grosse Holtforth and Castonguay 2005). It has, for instance, been found that clients’ rated satisfaction with assessment interviews was positively correlated both with clarity of assessment and with the option for participation in treatment planning. Interestingly, therapists’ rated satisfaction did not correlate with these negotiable variables (Eisenthal et al. 1983). Likewise, patients in psychiatric day care rated the treatment as more meaningful when they perceived the goals as clearly specified (Goldstein et al. 1988). Furthermore, in a study where quality of therapy was judged by independent assessors, one element that was found to be associated with what was perceived as “good therapy” was when the goals of the treatment were explicitly addressed by the client and the therapist (Hoyt 1980).

Tryon and Winograd (2002) published a review of studies on the impact on treatment outcome from two goal-related aspects in psychotherapy: *goal consensus*, which is to what degree there is a mutual agreement between the therapist and the client on the goals for therapy, and *goal collaboration*, which is to what degree the therapist and the client work conjointly with the goals for therapy. Tryon and Winograd identified 24 studies with relevance for assessing the impact of goal collaboration on treatment outcome and found considerable support for a relationship between these two variables. On goal consensus, they identified 17 studies generally favoring a positive relationship between this aspect and outcome, but to a lesser degree. In a subsequent meta- analysis on the subject, they included studies published from 2000 through 2009 (Tryon and Winograd 2011). Here, they identified 15 studies with a total sample size of 1,302 that yielded a goal consensus–psychotherapy outcome effect size of *r* = .34, a medium effect size, indicating that better outcomes can be expected when there is a consensus between patient and therapist regarding therapeutic goals and the processes to achieve these goals. When turning to the factor of goal collaboration, Tryon and Winograd identified 19 studies with a total sample of 2,260 patients that yielded a mean correlation of *r* = .33, indicating that psychotherapy outcome is enhanced when patient and therapist are actively involved in a collaborative effort to formulate goals for treatment.

The idea of the beneficial effects of formulating goals has also gained support from other areas of behavioral change. One study indicated that writing about life goals may be associated with health benefits, very much in the same vein as expressive writing about one’s past has been (King 2001). In student settings, goal pursuit has been positively associated with value endorsement, behavioral persistence, conceptual understanding, and personal adjustment, which in turn make a difference with regard to educational outcomes (Deci and Ryan 2000). When it comes to influencing human behavior, organizational and industrial psychology represents the area where the effects of goal setting has been most widely researched and where goal setting has been most consistently shown to have an effect on productivity outcome (Locke and Latham 2002). To summarize this brief overview on empirical studies, we can quite confidently state that there is an indication that the issue of personal goals and formulation of goals denotes an area of vital concern for human functioning and is of instrumental value in the process of influencing human behavior.

## The Definition of Goal as a Concept

### The Cognitive Position

Elliot and Fryer (2008) pointed to the ubiquity of goal-related constructs in psychology from both a theoretical and an empirical point of view. Despite ample use of the term *goal*, they noted that both researchers and theorists have neglected to offer a definition of the term and that it tends to be used as a rather vague concept where its meaning is taken for granted. Elliot and Fryer also stated that this lack of clarity is a grave concern, since it hinders progress in empirical research. A consensual definition was not to be found, but they drew some defining features of what is meant by a goal from the literature:

A *goal* is a cognitive representation of a future object that the organism is committed to approach or avoid. . . . A goal is focused on the future; it is a cognitive representation of something that is possible in the future. Goal-directed behavior is proactive, not reactive. It entails use of a future image as a guide to present behavior; it does not simply entail an immediate, unmediated response to a present stimulus . . . . Implicit in this conceptualization is that the mental image of the future possibility has a *causal* influence on present behavior. (Elliot and Fryer 2008, p. 245)

The aforementioned definition is very much in line with an earlier definition by Brunstein et al. (1998): “Personal goals are elaborate cognitive representations of what a person wants to achieve or avoid in his or her current life circumstances” (p. 494). In these definitions of the goal concept, a central tenet is the mental representation of a desired future outcome. These definitions might be unproblematic from a commonsense point of view. But when a definition rests upon another term, *cognitive representation*, this term needs to be defined to make sense of the first term. Trying to define the term *mental representation*, researchers run into a similar problem: the lack of a generally agreed definition. If they were to go along with Gallistel (2001), they could adopt the definition of a mental representation as “a system of symbols isomorphic to some aspects of the environment, used to make behavior-generating decisions that anticipate events and relations in that environment” (p. 9691). According to Tye (2000), representationalism appeals to the *transparency* of experience. The properties in a perceptual experience are presented as properties of objects perceived; they are not presented as mere properties of the experience itself. Thus, according to the representationalist view, the phenomenal character of an experience is due to its *representing* objective, nonexperiential properties. Thus, following this line of reasoning, goals are conceptualized as mental events that represent properties of the future, and these events can be considered to have causal influence on behavior.

### The Behavior-Analytic Position

In the behavior-analytic literature, references to goals or goal-directed behavior are less common. This should come as no surprise, since these concepts, in a dualist fashion, tend to rest upon hypothetical mental entities that are used for causal explanation of the individual’s behavior, a form of reasoning that is at odds with behavior-analytic thinking. However, the concept of goals is used in the literature in ways that imply that goals do have some bearing on the behavior of the behavior analysts themselves. For example, in the classic article by Baer et al. (1968), reference is made to the researcher’s goal. In the same vein, Wolf (1978) raised the question of the social significance of the specific behavioral goals that govern the applied work. Likewise, in the applied behavior analysis literature, goals are used as a concept that describes what regulates behavioral change programs (e.g., Cooper et al. 2007), and lately references have been made to the goals of contextual behavioral science as such (Hayes et al. 2012).

A notable exception within the behavior-analytic field is the organizational behavioral management (OBM) literature, where goals are frequently addressed, especially in terms of methodology. Different procedures of goal setting are described as a widely used and well-established method for increasing productivity (Tammemagi et al. 2013) and are often employed in combination with feedback procedures (Alvero et al. 2001). But in texts that deal directly with the basic behavioral terminology of goals, a picture much more colored by critical voices emerges.

Skinner (1985, 1989) stated that while words suggesting probable action seem to point to the future, they are in fact referring to past consequences. Intention is mainly a way to speak of operant behavior, and operant behavior is shaped by the histories of the subjects. The same goes for statements that human behavior is directed toward goals and the satisfaction of needs. These statements would translate into behavioral terms by saying that people behave in ways that have had reinforcing consequences in the past, and that these consequences have been made reinforcing through natural selection or operant conditioning.

There is no *current goal,* incentive, purpose or meaning to be taken into account. This is so even if we ask him what he is doing and he says, “I am looking for my glasses.” This is not a further description of his behavior but of the variables of which his behavior is a function; it is equivalent to “I have lost my glasses, I shall stop what I am doing when I find my glasses,” or “when I have done this in the past, I have found my glasses.” These translations may seem unnecessarily roundabout, but only because expressions involving goals and purposes are abbreviations. (Skinner 1953, p. 90)

This passage delineates two functions for behavior when talking about goals. One is that goals can be used as abbreviations, pointing out the past reinforcing consequences of behavior in a person’s environment; the other is that goals denote a consequence specified in advance that will terminate the behavioral event in question.

This way of talking about goals is quite in accordance with the analysis made by Baum (2005). In his words, the goals of an activity can be referred to as part of that activity. Goals are a quality of behavior rather than causes of behavior. For instance, in an individual’s behavior of walking toward the city center, the goal of walking (a specific location) is inseparable from the activity of walking. Walking to the beach would, in a functional sense, not be the same behavior with another goal. It would be a different behavior. This logic will inevitably make the same contingencies responsible for the goal as for the “goal-directed behavior,” since they are mere topographical aspects of the same behavioral entity.

On a more applied level, Fellner and Sulzer-Azaroff (1984) discussed goals and goal setting in an OBM context. They provided a general definition of goal setting as an established efficacious method for behavioral influence that specifies a level of performance toward which the person should work. In their account, goals are defined in terms of functional properties, and a goal will be seen as a stimulus that precedes behavior. When a verbal event—that is, a goal statement—reliably accompanies a reinforced response, this verbal event may acquire the property of a discriminative stimulus. Thereby, when uttered, this verbal statement will increase the probability of that response being emitted. By definition, this will mean that in the presence of such a goal statement, actual reinforcement is more likely, and in absence of that statement, it is less so. Furthermore, in their account they hypothesized that goal attainment may acquire the function of a conditioned reinforcer if goal attainment reliably accompanies the reinforcers that follow upon the actual behavior. From a commonsense perspective, the behavior-analytic conceptions of goals may seem like an odd entry, since the more usual way is to treat the goals of behavior as events that are separate from these behaviors and as events that are located in the future rather than in the history. The behavior-analytic conception, thereby, may pose problems with regard to the everyday use of the concept of goals.

While Fellner and Sulzer-Azaroff’s (1984) account may be satisfying the behavior-analytic task of making the processes studied accessible to experimental manipulation, it principally contains the possibility of substituting the goal statement with a random word, and the goal attainment with a token. Our assumption is that few would refer to the resulting behavior as goal directed. It is also hard to see how this use of the term *goal* would be helpful, even in the community of behavior analysts themselves when using the term in reference to their own enterprise. And what are behavior analysts to do with goals that are formulated about events that have no obvious counterpart in the individual’s experienced history—goals such as “world peace”? What are behavior analysts to do with verbally stated goals that are not a part of behavior that is directed at or under the control of the goals in question? After all, it is obvious that an individual can talk about walking toward the city center without acting accordingly, and thereby “failing to reach the goal.” Or if the individual’s goal is to reach the city center by foot, there may be several options for different behaviors that would be functionally equivalent in the sense that they would all make the individual end up at the same place. Yet, these options may differ in the efficiency of producing that outcome. There is even a possibility of talking about goals and behaving in a fashion that reasonably would be considered counterproductive to achieving those goals. It is easy to find objections to the behavior analytic passages above, since they seem to be incompatible with the usefulness of these words in everyday conversation.

At the same time, while these behavior-analytic ideas may defy the commonsense notion of a goal, they put a healthy focus on the task at hand, which is to formulate what seems to be a part of the subject’s future as a part of his or her learning history.

### Goals as Mental Representations

Representational conceptualizations of goals, on the other hand, seem to run smoothly with the everyday use of the term. When speaking of goals, the idea of a mental image of something that a human being wants almost seems like a perfect match with common sense. Individuals picture their lives in the future in a different, but more desired, state than today. These images may be expressed as verbal descriptions, and we will call these descriptions “goals.” Individuals are able to execute functional behavior in striving toward these goals, as long as the goals are deemed to be realistic.

But the problem pointed out by Elliot and Fryer (2008) is one of vagueness and lack of precision, which is a grave concern in scientific affairs. The problem is not its usefulness in everyday language, which in contrast seems rather unproblematic. As stated previously, the proposition was to treat goals as mental representations of the future events that can be considered causes of behavior. We will set aside all problematic aspects of the term *mental*, since this discussion is outside the scope of the present article. Even if one would accept the basic tenets of representationalism, according to Tye (2000), there will be a logical fallacy when treating goals as representations of future events. The future by definition has no objective features. It is no property of the external environment. If goals are representations, what do they *re-present*?

This would also mean that there is a basic problem with how goals, as representations, reasonably could be measured by standards of truth, realism, and correspondence to a world outside a mind. Further, it often stated both in commonsense reasoning and in the literature that it is desirable to set realistic goals (e.g., Cox and Klinger 2002). This would imply that one should avoid goals that are deemed to be unrealistic. But this distinction will also run into problems within a representational account. What are the real-world properties these goals could be judged against? They can form no part of the future but only be a part of the past or present. And unrealistic goals that have no correspondence in the history of man (e.g., “world peace”), and sadly no realistic outlook of being achieved in a foreseeable future, may yet inspire people to do greats deeds. These could be deeds that would be valuable to the person and to other human beings, in spite of not ultimately terminating warfare. On a less global scale, it is easy to imagine similar scenarios regarding health-related goals on an individual basis (“live ’til I get hundred”). It is hard to fit goals into the representationalist view, since there is no possibility to determine what the goals are *re*-presentations of. Etymologically, the prefix *re* carries with it the meaning of “again,” which makes sense to the idea of re-presenting the past. Future goals should, if anything, rather be *pre*-presentations.

## A Behavior-Analytic Account of Goal-Directed Behavior

### The Task for Behavior Analysis When It Comes to the Concept of Goals

The first question before we continue to delineate a behavior-analytic account of what it means to have a goal is why we should do it. As pointed out by Moore (2001), there is a challenge for behavioral theorists to make sense of psychological terms that appeal to the mental. When attempting to understand the usage of a term such as *goals*, one needs to look closer at verbal behavior. And to deepen that analysis, one needs to understand multiple controls of verbal behavior. As Moore stated, one can assume that this behavior is controlled (a) partly by sociocultural traditions and (b) partly by operations and contact with other aspects of the environment. People will talk about goals because a verbal community has reinforced this behavior, but also because this verbal behavior has been functional with regard to actions the person has taken in a particular context. Terms such as *goals* may be perfectly reasonable as descriptive terms relating to the probability of a response. Or as put by Foxall (2004): “The testimony that people give us about their intentions, plans, hopes, worries, thoughts and feelings is by far the most important source of information we have about them” (p. 112).

The research cited earlier indicates that the verbal behavior referred to as goal statements may be useful for the purpose of both predicting and influencing behavior. And as already indicated, behavior analysts use the term *goals* in a way that hints that they themselves also seem to have goals and find use in formulating them. The analytic approach of behavior analysis is fundamentally practical. In this vein, when Fellner and Sulzer-Azaroff (1984) pointed to the behavior-analytic objective in avoiding mentalist conceptions, this practical ambition corresponds in an OBM context to the search for concepts that has a value in guiding managers and administrators toward the environmental conditions that may be influenced. Rather that, than attempting to influence inaccessible mental processes in employees.

### Goal-Directed Behavior as Rule Governed

A reasonable way to study goals would be to ask people about them in one way or another. Essentially, that is how the empirical data on goals, cited previously, have been collected. The fact that goal-setting procedures tend to be stressed in the OBM literature and in clinical literature should be considered from the perspective that both areas primarily deal with verbally competent individuals. In these areas, both the behavior under scrutiny and the influence it is exposed to are often verbal. So, in order to provide further precision in the analysis, the events under study are considered to be verbal responses—that is, goal statements—rather than goals per se (O’Hora and Maglieri 2006).

To follow Skinner in an analysis, goal statements are a form of human behavior that may in turn influence other behavior. Talking about goals is one functional class of behavior, and behavior under the influence of such talking is another functional class. When focusing on what effects goals have on human behavior overall, what should be analyzed is not a goal as an object but the relationship between these two functional classes of human behavior. It is thus a behavior–behavior relationship (Hayes and Brownstein 1986; Leigland 2014). If a person states a goal and then acts to achieve it, he or she is interacting with his or her own behavior in the moment, not with a future object.

Describing future goals in the present as related to events that are hitherto not directly experienced touches on Skinner’s (1966) analysis of rule-governed behavior. Skinner did not use this term with regard to *goals,* but it is easy to see its relevance for our subject area. A rule that influences subsequent behavior is, according to Skinner, an antecedent specifying behavior and consequence. The idea of treating goals as rules or statements specifying contingencies, along with treating them as motivating operations rather than as discriminative stimuli, has by and large been adopted by the OBM literature (Agnew and Redmon 1993; Malott 1993).

An example would be a man looking for his glasses in an entirely new way and place upon hearing “Dig into the pot of mashed potatoes and you will find your glasses!” Or, to use an example of more complex behavior, “Join us in the demonstration next Saturday, and you will make a contribution to world peace!” If someone then takes part in the demonstration to achieve this end, it would be a case of rule-governed behavior. It is also quite reasonable to expect that within a cognitive framework, this behavior would be called “goal driven,” where the goal that drives the behavior would be conceptualized as a mental image of future possibilities. But in Skinner’s analysis, this goal is neither mental nor future. It is behavior under the control of the past and present context of the listener to the rule.

We argue that it is quite possible to make a sensible and parsimonious statement regarding goal-directed behavior from a behavior-analytic position. Goal-directed behavior is behavior under the influence of other behavior—that is, verbal behavior or, more specifically, goal statements. In the simple case, the establishing of a rule or instruction is overt verbal behavior. But it may equally well be covert. The problem with this position, when it comes to the concept of goal-directed behavior, is not at the basic conceptual level. The problem is what Skinner left unanswered: the question of how this verbal behavior can influence other behavior.

### A Relational Frame Theory Account of Goal-Directed Behavior

In order to further the understanding of the behavioral processes underlying goal-directed behavior, O’Hora and Maglieri (2006) extended the analysis by incorporating principles from relational frame theory (RFT; Hayes et al. 2001; Törneke 2010). The rationale for this extension is twofold: It should enable increased precision, and it should rest upon basic behavioral principles amenable to empirical investigation. But O’Hora and Maglieri also noted that while behavioral conceptualizations of goal setting have attempted to account for putative behavioral histories, they have ignored much of the empirical research on the effects of goals and goal setting that is described in the cognitive literature. These effects must be accounted for in a comprehensive behavioral conceptualization of goal-directed behavior.

From an RFT perspective, it is suggested that verbal behavior is a special way of responding to relations between stimuli (Törneke 2010). By means of operant learning, humans acquire this ability to respond to socially established contingencies early on (Healy et al. 1998; O’Hora et al. 2004). Technically, this ability is called “arbitrary applicable relational responding” or, more metaphorically, “relational framing.” It is arbitrary in the sense that humans learn to relate events based on arbitrary contextual cues rather than just on their physical characteristics (Hayes et al. 2001). In RFT, the analysis of thinking follows that of Skinner in treating thinking as private verbal behavior under contextual control. Thus, private behavior can acquire functions as a result of the individual’s way of relating events to each other rather than purely on that individual’s direct experience of these events.

Different ways of relating under contextual control have been theoretically identified and experimentally verified. This research has covered relations of coordination—that is, relating one event as the same as or equivalent to another relations of opposition (Barnes-Holmes et al. 2004a; Carpentier et al. 2003; Dymond et al. 2008; Roche and Barnes 1997)—as well as comparative relations—that is, relating events as more-than or less-than (Barnes-Holmes et al. 2004c; Berens and Hayes 2007; Dymond and Barnes 1995; Munnelly et al. 2010). Other types of relations are temporal relations (O’Hora et al. 2008), causal relations (Dack et al. 2009), and hierarchical relations—that is, responding to one event as belonging to another, or being part of or being included in another category of events (Gil et al. 2012). Yet another type of framing is the ability to take a certain perspective, or deictic relations (McHugh et al. 2004; Weil et al. 2011).

These relations, established by arbitrary contextual cues, alter the way events or stimuli acquire functions for further behavior. Among the functions that have been studied experimentally are respondent conditioning (Dougher et al. 1994, 2007), mood (Barnes-Holmes et al. 2004b; Cahill et al. 2010), and evaluative conditioning effect (Valdivia-Salas et al. 2013). These functions imply that if one is capable of interacting with arbitrary contextual cues in a way that transforms stimulus function, words or gestures may provide cues that can alter the functions of stimuli. These stimuli thereby acquire new “meaning.” The statement that “X is better than Y” contains the word “better” (cuing comparative framing), which may transform the valence of a not-yet-experienced event. Simple examples of comparative framing would be the statements “Japanese whisky is better than Scottish,” “German cars are better than French ones,” and “Corn-fed chickens are better than ordinary ones.” Given that the listener has experienced drinking Scottish whisky, driving French cars, and eating ordinary chicken, he or she may now relate to Japanese whisky, German cars, and corn-fed chicken as more appetitive, without any direct contact with these stimuli. These processes may be accomplished as a result of the listener having the repertoire of interacting with arbitrary contextual cues in a way that transforms the stimulus functions of present events. These repertoires can be shaped, under contextual control, to increasing complexity (Barnes-Holmes et al. 2004a; Lipkens and Hayes 2009). Once the basic repertoire is available, quite complex actions can take place.

### Goals as Relationally Framed Events

For the present discussion on goal-directed behavior, temporal framing is of special importance. All organisms have the ability to interact with changes in the environment. But once the stimulus functions of the environment transform as a result of arbitrary contextual cues, a new route to behavioral change is established. Just as stimuli can be acted upon as “better” without any direct experience, they can be acted upon as “coming later” without any prior experience of the stimuli or the events occurring in this specific order. So, through the processes described, a person will be able to interact with verbal stimuli so that he or she will constitute a rule in the form “If you do Z, you may acquire X, which is better than Y.” If the rule influences the following behavior, the result is what is typically called “rule-governed behavior.” In this way, RFT offers an analysis of how a rule can specify behavior and consequence, to use Skinner’s (1966) words.

O’Hora and Maglieri (2006) have incorporated the conceptual framework of RFT in the analysis of goal statements as a means of understanding the process of influencing behavior in an organizational context. They pointed out a limitation in the behavioral literature, when it often neglects empirical findings from other areas in the field and may fail to offer an account of these findings. Specifically, they pointed out that research unanimously shows that when goal difficulty increases, performance improves, regardless of whether the goal is attained or not. These results run counter to the idea of goal statements as discriminative stimuli. Increased difficulty would reasonably be associated with less likelihood of reinforcers to occur (i.e., goal attainment), and performance should be expected to decrease.

But O’Hora and Maglieri argued that effective goal statements function by specifying a level of performance that will relate feedback statements over ongoing performance, in a “more/less-than” frame, to the behavior defined by the goal. If the goal is to complete 10 activities, stating that one has completed three activities rather than none relates the ongoing performance to the goal statement. The approaching of the defined goal level is only achieved indirectly, by verbal means, but will allow for the reinforcement of “being on the right way.” Thus, reinforcement is quite possible, even though the goal in itself is not attained. In this way, the behavior in question acquires derived reinforcing functions through a verbal statement signaling closer proximity toward an event or state not yet reached. But in order for this function to persist over time, the relation between the goal level of performance and the reinforcement inherent in attaining the goal needs to be maintained, to retain the reinforcing effects of verbal feedback. Behaviors that are task-relevant will be more likely to occur and those that are task-irrelevant will be less likely, when goal and feedback statements are provided. O’Hora and Maglieri (2006) also touched upon a more process-related feature of goal setting in production facilities when relations are derived “based on the deictic (I-You) relations between each employee’s view of him or herself and his or her view of other employees (e.g., ‘We’re all in the same boat’)” (p. 154). In this way, RFT can be used in an analysis of goal-directed behavior as behavior under the influence of other behavior and to analyze how antecedent and consequential functions of events change owing to derived relational responding.

## Implications of a Behavior-Analytic Account of Goal-Directed Behavior

It is easy to agree with the previously mentioned point made by Elliot and Fryer (2008) that lack of generally agreed concepts is an obstacle for scientific progress in a field. The concept of goals as a determinant of human behavior represents such a field. The traditional way to conceptualize goals, as mental representations that govern behavior, is so well established that approximations of this view can be used with ease in everyday conversation. We have argued that treating goals as mental entities is unpractical when searching for a more precise formulation. Despite the relative paucity of a theoretical elaboration of the goal concept within earlier behavior-analytic thinking, we find it quite possible to make a sensible definition of goal-directed behavior from this position. RFT may serve as a basis for such a definition, and it may also be used to provide increased precision in an analysis of goal concepts. So, to summarize our position, we treat goal-directed behavior as behavior under the influence of other behavior—that is, verbal behavior, or rather, the more specific instance of goal statements. Goal statements will function as rules—in other words, antecedents specifying behavior and consequence. These functional relationships are under the control of the past and present context of the listener to the rule, and they may be analyzed in terms of how antecedent and consequential functions of events change as a result of derived relational responding. The latter will be especially applicable when goal statements denote consequences that have not been directly contacted.

A behavior-analytic approach should also be practical. In the present paper, we focus on the impact of goals for variables concerning health or quality of life and the goal-formulating process that is part of therapeutic work. Needless to say, the practical usefulness of goal concepts should be of central importance in these areas.

### Goal Statements Predict Well-Being

As we have concluded earlier, endorsing certain types of goal statements correlates with health and well-being (e.g., Brunstein 1993; Kasser and Ryan 1993, 1996; Williams et al. 2000). This correlation could reflect a simple relationship between certain goal statements and behavior that ultimately increases the likelihood of contacting the reinforcers specified in a rule. Some goal statements are more likely to be associated with behavior that leads to well-being. But apart from the discriminative functions of this kind of verbal behavior, goal statements will also establish derived functions that are potentially appetitive (or aversive). These functions are contacted by framing events using comparative, temporal, and causal frames (“If I do X now, I will be in a better position to attain Y later”).

In the appropriate context, this framing can be done without any direct contact or experience with the events in question. The so-called future is verbal behavior in the present, and different stimulus functions are established by relational framing. From a phenomenal point of view, it should be noted that by using the concept verbal behavior, we by no means argue that the apparent future is necessarily perceived as constructed by words only. Images may also be an important part of the experience. It is as if the individual can see the future, in the same sense described by Skinner (1953) as seeing “stimuli which are not present” (p. 266). In this case, it would be seeing under the control of derived relations between events. It is quite reasonable that in some instances just formulating future goals can affect individuals’ emotional state and thus make them feel better (King 2001), just as thinking about the future can make them feel bad.

A similar analysis will clarify why a predominance of avoidance goals tends to be associated with increased indices of psychopathology (Grosse Holtforth and Grawe 2002). If the behavior of making goal statements is under a predominantly aversive control (“If I do X when Y, I may avoid the aversive Z later”), that verbal behavior will increase indirect contact with the aversive event to be avoided—for example, anxiety. In this situation, these goal statements will relate a multitude of events in the ongoing environment to the eventuality of experiencing anxiety and thereby potentially increase the perceived aversiveness of that environment, a process that may ultimately fuel anxiety. So, apart from the possibility that goal statements may be instrumental in behavior–behavior relationships, where the latter behavior ultimately steers the individual to good or bad health, they may set the individual in emotional contact with appetitive or aversive aspects of the world through the derived functions of this world. This process could be considered an important aspect of the motivative functions of goal statements.

### Life Goals

The importance of successful goal-directed behavior could, of course, be equated with behaviors that ultimately lead to consequences verbally specified in advance. But this effect does not necessarily imply that this instance is the only one where behavior analysts are able to identify the beneficial effects of goal statements. A question that becomes critical when it comes to the term *unrealistic goals*. A behavior analytic account does not rest upon representational, realist assumptions, and therefore the usefulness of goal statements will not be dependent on an assumed correspondence between the content in the goal statements and a world different from where these statements were made.

We could treat goal-talk as any behavior—in other words, as having the potential to serve a multitude of functions. This means that goal statements may be adaptive or maladaptive in promoting health-related activities or behaviors that are important to the community, regardless of these behaviors not necessarily guaranteeing an outcome such as old age or world peace. In approaching goal-directed behavior as a behavior–behavior relationship, behavior analysts cannot make any a priori assumption that one behavior (goal-talk) influences the other behavior. This question will always be an empirical one. From a behavior analytic point of view, the distinction between goals as intrinsic or extrinsic (Kasser and Ryan 1993, 1996) may not be very meaningful in itself. Actually, this discussion parallels the discussion of intrinsic or extrinsic reinforcers, where some researchers have promulgated the former ones as superior in many ways, while the basis for making this distinction has been severely criticized by others (Dickinson 1989). Goal statements falling in the two descriptive categories of intrinsic and extrinsic could simply be seen as reflecting different categories of potentially reinforcing properties of the environment, as well as rules how to contact these reinforcers.

The relation between the level of performance defined by the goal statement and external reinforcement should be maintained in order for the derived reinforcing effects of verbal feedback to function over time. In goals referred to as intrinsic, it would probably be harder to uphold the reinforcing relation contained within a less-than frame, in the way described by O’Hora and Maglieri (2006). But it would be reasonable to expect such a goal orientation to be associated with behaviors that may contact a wide range of reinforcers pertaining to affiliation, well-being, and community contribution—that is, predominantly social reinforcers. If theorists are speaking in terms of rule-governed behavior, this phenomena would be considered in terms of *augmentals* (Törneke 2010). An augmental is a rule that affects the degree to which different consequences function as reinforcing or punishing. It can also be described as a form of verbal establishing or motivating operation. These goals may serve important functions, irrespective of whether the state described in the goal statement will be reached. It may be worthwhile striving for old age and peace, even if the person may not live long or warfare not terminated.

### The Incremental Effects of Goal Statements in Clinical Work

Collaboration and consensus over treatment goals have been found to have beneficial effects in clinical work (Tryon and Winograd 2002, 2011). This finding fits well with the preceding analysis, as goal statements affect to what extent consequences will function as reinforcers of the behavior in question—or put differently, how goal statements may augment functions of directly contacted consequences. For example, the goal of exposure-based treatment may be formulated as the ability to live a more independent life, less controlled by anxiety. Steps taken in the treatment and their consequences may now be framed in hierarchy with this ultimate treatment goal and thereby be reinforcing, even though the individual’s life might still be largely a dependent one and controlled by anxiety.

Goal statements may provide stimulus functions that reduce the likelihood of off-task and counterproductive behavior (O’Hora and Maglieri 2006). In treatment, formulating goals could imply lessening the appetitive features of various types of avoidant behaviors, just by pointing out the incompatibility between these strategies and the chosen goals. When a goal is defined not as a mental entity but as verbal behavior, it is conceptualized as a variable that readily lends itself to manipulation. This kind of manipulation is what clinicians would do in verbal therapy when engaging in goal collaboration. The beneficial effects of goal-talk in therapy could be understood as a mutual process, where two (or more) persons bring their behavior under the control of shared verbal behavior—that is, goal statements.

Goal-talk may not only serve a useful purpose in orienting mutual future behavior in a way that is more likely to reach the consequences specified, but also promote a sense of communion between those involved. It may foster cohesion in therapeutic work and ultimately be instrumental in creating a working alliance beyond specifying outcome or outcome domain. Sharing goals will allow for an experience of “we-ness” based on a deictic (I-You) frame, thereby establishing one’s role with regard to the other (Vilardaga and Hayes 2010). The process of establishing and maintaining a therapeutic alliance is considered pivotal in therapy, and agreement on goals is an important part of this working alliance (Horvath et al. 2011). It is reasonable to expect the mutual formulation process to be associated with perceived therapeutic qualities.

As stated earlier, goal collaboration and agreement tend to have a general positive correlation with outcome. One aspect of this correlation is probably that reinforcers specified by rules are more effectively attained when the process of formulating goal statements is emphasized. Another aspect may equally well be in terms of the therapeutic relationship. Goal statements made by clients provide abbreviations of their behavioral histories (Skinner 1953). Attending to these goals will be attending to the client as a historical being and thereby contributing to a process beneficial for the therapeutic alliance.

Another common therapeutic situation is work with clients showing deficiencies in their capability for goal-directed behavior more generally. Based on RFT, the present conceptualization offers a number of entries for analyzing and training the core behavioral processes that constitute complex behavior, similar to those exemplified elsewhere (Rehfeldt and Barnes-Holmes 2009). This kind of training could include training a person in specific relational repertoires such as, for example, temporal, deictic, and hierarchical frames. Ultimately, this training would focus on rule-governed behavior and especially the form called tracking (Törneke 2010).

If goal-directed behavior is conceptualized as behavior under the influence of verbal behavior, defined as relational framing, we see no hindrance for the empirical findings cited earlier in this article to be assimilated within a behavior-analytic model. This line of thought has potential for analyzing goal-directed behavior without falling prey to concepts that are not readily amenable to either observation or deliberate influence. RFT offers a conceptual account in which goals and the problem of pre-presenting the future in a parsimonious way can be linked to a conceptual apparatus and psychological processes that readily lend themselves to empirical investigation. As argued previously, the experimental work within the RFT tradition offers a fruitful basis for investigating the processes that constitute the capacity for formulating goal statements and orienting one’s behavior in accordance with these statements. The goals for that analysis should be increased precision and an opening for new ways to understand and influence behavior. The way to go there is to consider goals not as mental but as environmental events. Goals are a type of behavior that occurs in the same environment as other behavior.
